# Osteoid Osteoma of the Hallux: A Diagnostic Challenge

**DOI:** 10.5334/jbsr.2497

**Published:** 2021-06-11

**Authors:** Aspara van Straaten, Stefan Clockaerts, Filip Vanhoenacker

**Affiliations:** 1 AZ Sint-Maarten, Mechelen and University (Hospital) Leuven, BE; 2 AZ Sint-Maarten, Mechelen and University (Hospital) Antwerpen/Ghent, BE

**Keywords:** computed tomography, magnetic resonance imaging, osteoid osteoma, hallux

## Abstract

**Teaching point**: Unexplained bone marrow edema on MRI warrants further investigation with CT to demonstrate a nidus which is pathognomonic for an osteoid osteoma.

## Case Presentation

A 45-year-old man presents with progressive, disabling pain at the left hallux for the past five months. There is no history of trauma, fever, or rheumatological disease. Physical examination shows a swollen and painful toe, without redness. Neurovascular examination and functional testing are normal. Analgesics, including as non-steroidal anti-inflammatory drugs (NSAID) do not improve pain.

**Figure 1:**
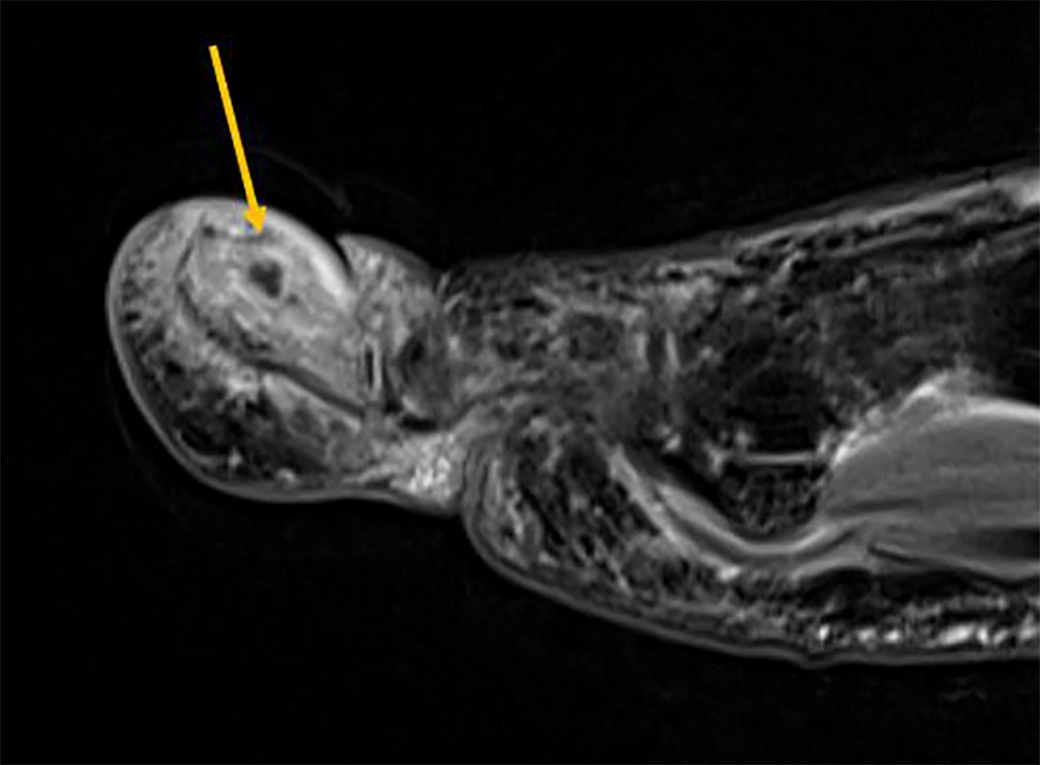


**Figure 2:**
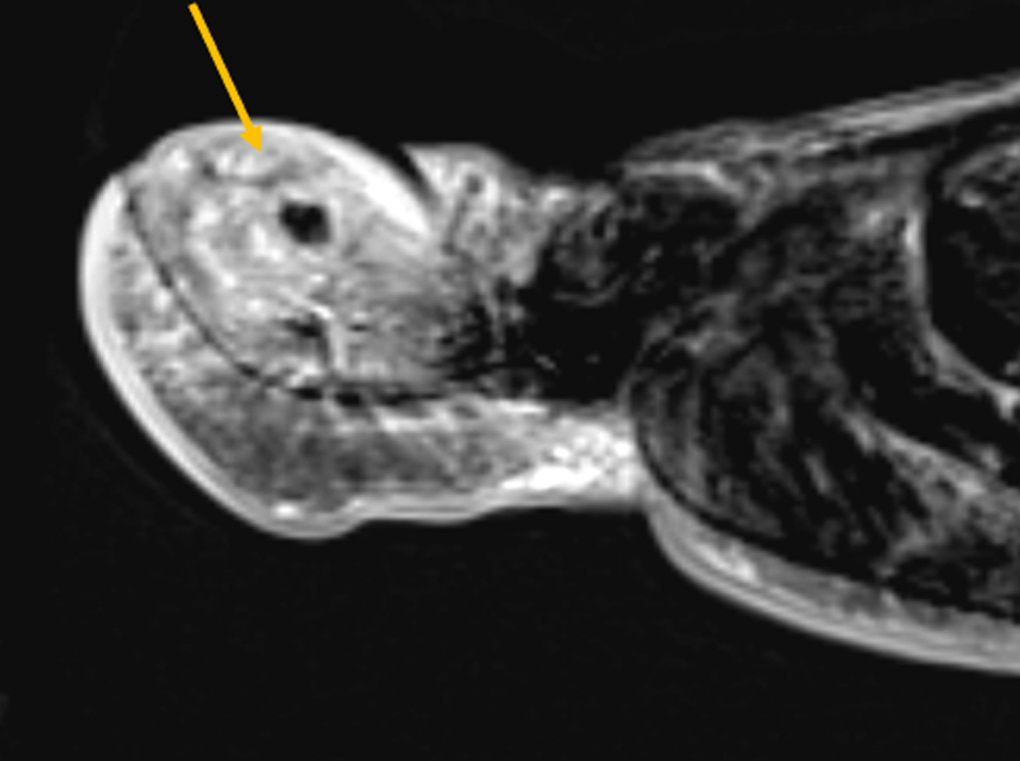


**Figure 3:**
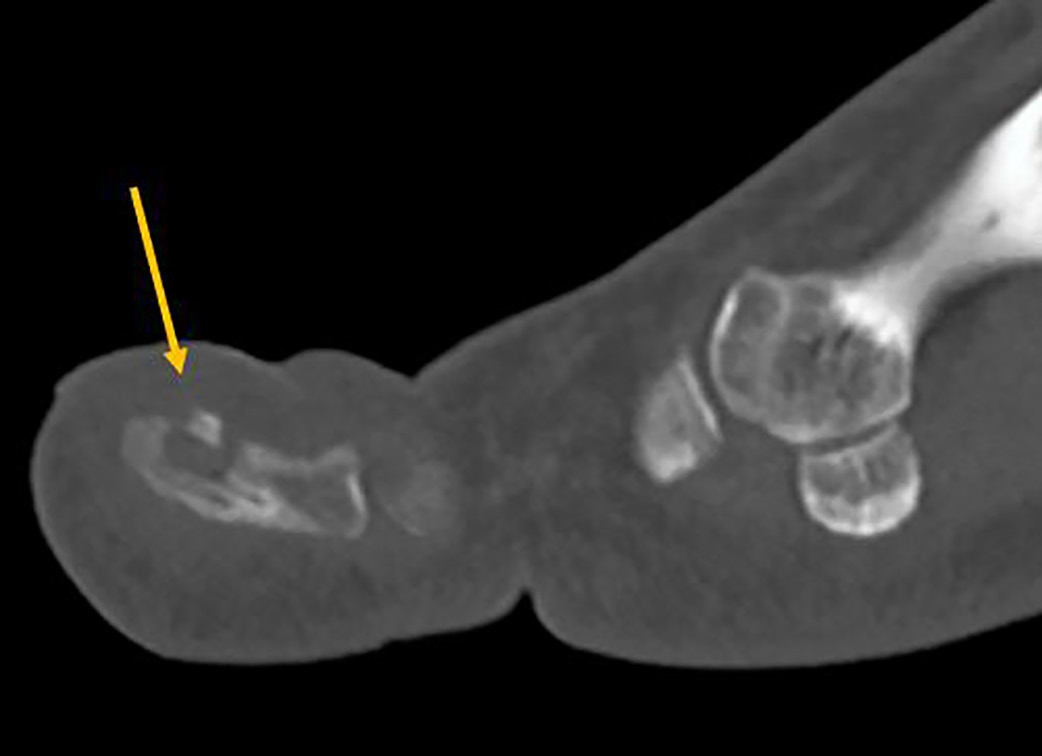


Conventional radiography is unremarkable. MR Imaging shows enhancing bone marrow edema of the distal phalanx and subtle accompanying soft tissue edema. There is a small T2-hypointense focus (***Figure 1***, sagittal fat suppressed [FS] T2-weighted image, arrow) in the dorsal cortex of the distal phalanx, showing absence of enhancement (***Figure 2***, sagittal subtraction image before and after gadolinium contrast, arrow). Subsequent computed tomography (CT) examination shows a cortical radiolucency with intralesional dense focus (***Figure 3***, sagittal reformatted image, arrow), in keeping with a calcified nidus of an osteoid osteoma (O.O.).

## Discussion

Osteoid osteoma is a rare and benign bone tumor that occurs mainly in children and adolescents with a peak incidence between the first and third decades. It comprises 10% of all benign bone tumors. Only 2% of O.O. are located in the distal phalanx of the toe [1]. Most patients present with a swollen toe and nocturnal pain responding typically to NSAID, although this may be absent.

Radiography may demonstrate a subtle cortical radiolucent nidus with variable degree of intralesional calcification and surrounding reactive sclerosis [1]. Because of the small size of the nidus and the absence of reactive sclerosis, radiography may be unremarkable in the early stage of the disease. MRI may demonstrate extensive bone marrow edema in the involved bone as well as soft tissue edema, but the nidus may be difficult to demonstrate. In the latter scenario, the diagnosis may be challenging on MRI as the etiology of bone marrow edema may be unrevealed.

Therefore, CT scan is often required to demonstrate the nidus unequivocally and may be regarded as the preferred imaging modality [1]. The imaging diagnosis may include chronic osteomyelitis with formation of a sequestrum, but the absence of fever and discoloration of the skin argues against this diagnosis.

The therapy of O.O. of the phalanges consists of excision of the lesion by open surgery [1]. Although percutaneous CT-guided radiofrequency or laser ablation is currently the standard treatment for O.O. on other locations, for superficially located lesions such as the hallux, there is a risk of thermal necrosis of the skin. In addition, excision allows definitive histological proof and exclusion of alternative diagnosis.
